# 

**Published:** 1899-07

**Authors:** 


### BOOKS RECEIVED.

Transactions of the Wyoming State Medical Society. First and Second Annual Meetings, held at Rawlins and Rock Springs, May and November, 1898. E. Stuver, M. D., Secretary. Denver: App Engraving and Printing Company. 1899.

The Diseases of the Nervous System. A Text-book for Physicians and Students. By Dr. Ludwig Hirt, Professor of the University of Breslau. Second American edition, revised from the second German edition by August Hoch, M. D., assisted by Frank R. Smith, A. M. (Cantab.), M. D., Assistant Physicians to the Johns Hopkins Hospital. With an Introduction by William Osler, M. D., F. R. C. P., Professor of Medicine in the Johns Hopkins University. Octavo, with 178 illustrations. Cloth, $5.00; sheep, $6.00. Sold only by subscription. New York: D. Appleton & Co. 1899.

Twentieth Century Practice. An International Encyclopedia of Modern Medical Science. By leading authorities of Europe and America. Edited by Thomas L. Stedman, M. D., New York City. In 20 volumes. Vol. XVI., "Infectious Diseases." New York: William Wood & Co. 1899.

Transactions of the American Association of Obstetricians and Gynecologists. Eleventh Annual Meeting, held at Pittsburg, September 20, 21 and 22, 1898. William Warren Potter, M. D., Secretary. Philadelphia: Wm. J. Dornan, Printer. 1899.

Transactions of the Twentieth Annual Meeting of the American Laryngological Association. Held in the City of Brooklyn, May 16, 17 and 18, 1898. Henry L. Swain, M. D.; Secretary. New York: D. Appleton & Co. 1899

International Clinics. A Quarterly of Clinical Lectures on Medicine, Neurology, Surgery, Gynecology and Obstetrics, Ophthalmology, Laryngology, Pharyngology, Rhinology, Otology and Dermatology, and Specially Prepared Articles on Treatment and Drugs. By professors and lecturers in the leading medical colleges of the United States, Germany, Austria, France, Great Britain and Canada. Edited by Judson Daland, M. D. (Univ. of Penna.), Philadelphia, Instructor in Clinical Medicine and Lecturer on Physical Diagnosis in the University of Pennsylvania; Assistant Physician to the Hospital of the University of Pennsylvania, etc.; Fellow of the College of Physicians of Philadelphia. Volume I. Ninth series. 1899. Octavo, pp. x.—303. Philadelphia: J. B. Lippincott Co. 1899.

A Manual of Surgical Treatment. By W. Watson Cheyne, M. B., F. R C. S., F. R. S., Professor of Surgery in King's College, London, Surgeon to King's College Hospital, etc; and F. F. Burghard, M. D, and M. S. (Lond.), F. R. C. S., Teacher of Practical Surgery in King's College, London, Surgeon to King's College Hospital, etc. In six imperial octavo volumes, with illustrations. Vol. I., 285 pages, with 66 illustrations. Cloth, $3 00 net. Philadelphia and New York: Lea Brothers and Co. 1899.

Progressive Medicine, Vol. II. A Quarterly Digest of Advances, Discoveries and Improvements in the Medical and Surgical Sciences. Edited by Hobart Amory Hare, M. D., Professor of Therapeutics and Materia Medica in the Jefferson Medical College of Philadelphia. Octavo, handsomely bound in cloth, 472 pages, 56 illustrations and 3 full-page plates. Philadelphia and New York: Lea Brothers & Co.

Transactions of the American Dermatological Association at its Twenty-second Annual Meeting, held in Princeton, N. J., May 31 and June 1, and in New York City, June 2, 1899. John T. Bowen, M. D., Secretary. Concord, N. H.: The Rumford Press. 1899.

Public Health Reports. Issued by the Supervising Surgeon-General Marine Hospital Service, under the national quarantine act of April 29, 1878, and the act granting additional quarantine powers and imposing additional duties upon the Marine Hospital Service, approved February 15, 1893. Vol. XIII. Nos. 1 to 52. Washington: Government Printing Office. 1899

Electro-Hemostasis in Operative Surgery. Being a work supplementary to the Author's Treatise on the Diseases of Women. By Alexander J. C. Skene, M. D., LL. D., Professor of Gynaecology in the Long Island College Hospital, Brooklyn, N. Y. Octavo, pp. x—173, illustrated. New York: D. Appleton & Co 1899.

Atlas on Fractures and Dislocations. By Prof. Dr. H. Helferich, of Greifswald. Translated from the third German edition by J. Hutchinson, Jr., F. R. C. S. Sixty-eight chromo-lithographic plates with descriptions, and 130 pages of treatise, illustrated by 126 woodcuts. Wood's Series of Medical Hand Atlases. Muslin, $3.00 net. Wm. Wood & Co. 1899.

Defective Eyesight. The Principles of Its Relief by Glasses By D. B. St. John Roosa, M. D., LL.D., Professor Emeritus of Diseases of the Eye, New York Post-Graduate Medical School and Hospital; Surgeon to the Manhattan Eye and Ear Hospital, etc. 12mo, pp. x—193. Illustrated. New York: The Macmillan Company. 1899.

Hay-Fever and Its Successful Treatment. By W. C. Hollopeter, A. M., M. D., Clinical Professor of Pediatrics in the Medico-Chirurgical College of Philadelphia; Physician to the Methodist Episcopal Hospital, etc. Second edition, revised and illustrated. Price, $1.00. Philadelphia: P. Blakiston's Son & Company. 1899.

Practical Diagnosis. The Use of Symptoms in the Diagnosis of Disease. By Hobart Amory Hare, M. D, B. Sc, Professor of Therapeutics and Materia Medica in the Jefferson Medical College of Philadelphia. Fourth edition, enlarged and thoroughly revised. In one octavo volume of 623 pages, with 205 engravings and 14 full-page colored plates. Cloth, $5.00 net. Philadelphia and New York: Lea Brothers & Co. 1899.

---

### LITERARY NOTES.

### INDEX TO VOLUME XXXVIII.

PAGE.

ABORTION . . . . . . . . . . . 533
Absorption and Excretion of Iron . . . . . . . . . . . . . . . 685
Accommodation in Lower Animals . 600
Acetylene, Toxic Action of . . . . 371
Gaslight for Examination of Eyes . . . . . . 522
Acne Vulgaris and Rosacea, Electricity in . . . . . . . . . . . . . 254
Acting Assistant-Surgeons, Examination of . . . . . . . . . . . 301
Age, Sex and Race in Surgical Affections . . . . . . . . . 51
Albuminuria in the Puerperium . . . 692
Alcohol, Influence of on Digestion . 370
Alimentary Canal, Breaking Up of Fat in . . . . . . . . . . . 200
Alopecia Areata . . . . . . . . . 276
Alumni Reunion, Address by Z. J. Lusk . . 747
Address by W. G. Taylor . . 748
Address by William Warren Potter . 750
Amblyopia, Case of Quinine . . . . 279
Anasin . . . . . . . . . . . . . 291
Anesthesia, Local . . . . . . . 296
Anesthesia, to Prevent Nausea After 206
Aneurism of the Aorta . . . . . . 53
Appendicitis in Children . . . . . . 599
Argon, Vegetation With and Without . . . . . . . . . . . . . . 202
Argonin in Middle Ear Catarrh . . . 374
Army and Navy Notes . . . . . 59, 135
Physicians from Buffalo . . 136

BABCOCK, C. E. P., Camp Sewers . . . . . . . . . . . . 591
Battle of July Third . . . . . . . 66
Beef Tea as a Food . . . . . . . . 286
Benedict, A. L., Hyperchlorhydria . . 823
Benzosol in Diabetes Mellitus . . . 293
Bicyclists, Death of Two . . . . . 135
Bile Tract Operations, Incision for . 51
Bishop, Edwin R., Smallpox in Western New York . . . . . . . . 430
Blood, Abnormalities of . . . . . . 481

PAGE.

Books Received . . . . 77, 156, 237, 317, 398, 477, 558, 638, 718, 804, 878, 950
Boric Acid, Influence of on Nutrition, 370
Brain Anatomy, Rules of . . . . . 763
Brown, Ira C., Assignment of . . . 136
Buffalo as a Recuperating Station for Soldiers . . . . . . . . . . 137
Burns, A Plaster for . . . . . . . 206
Cause of Death After . . . . 206

CAMP BLACK, Inspection of . . 222
Sewers . . . . . . . . . 591
Water Supply . . . . . . 592
Camps of Instruction . . . . . . . 492
Cancer, Uterine—Early Diagnosis . . 576
Carcinoma of the Axilla . . . . . . 53
Testicle . . . . . 23
Carotid, Clamping of . . . . . . 53
Casualties in the War with Spain, 138, 220
Cataract in Glass Blowers . . . . . 213
Catheters and Cystitis . . . . . . . 757
Cerebro-Spinal Fluid . . . . . . . 201
Christian Scientists, a Good Place for, 380
Cholera Bands . . . . . . . . . . 63
Infantum . . . . . . . . . 409
Treatment of . . . 20
Cholelithiasis in Infancy . . . . . 595
Pathogenesis of . . . 832
Chorea, Treatment of . . . . . . . 449
Clark, Edward, Disposal of Garbage, Sewage, etc., in Camps . 580
L. Pierce, Hysteria Associated with Epilepsy . . . . . . 5c2
Clemesha, J. C., Case of Myxedema, 179
Case of Quinine Amblyopia . . . 279
Clinton, Marshall, Impressions of Cuba and the Cubans . . . . . . 816
Colles's Fracture . . . . . . . . . 571
Comforts for the Sick and Wounded, 65
Concho, The, and Harper's Weekly, 137
Conjunctiva, Bacteria in the Normal . . . . . . . . . . . . 214
"Conservative Surgery" as Applied to the Uterus and Appendages . . 721
Copper, Physiological Action of . . . 202
Cornea, Opacity of . . . . . . . . 602

PAGE.

Corner-stone Ceremonies, Medical Society County of Kings . . . . 382

**Correspondence:**

Four Cases of Placenta Previa, O. C. Hammond . . . . . 608
Osteopathy vs. The Medical Age, William M. Warren . . . . 375
Service at Fort McPherson, Ga., John Hudson Grant . . . . 217

Cough, Treatment of in the Phthisical . . . . . . . . . . . 532
Craig Colony Prize . . . . . . 450
Creosote in Phthisis Pulmonalis 690
Cuba and the Cubans, Impressions of, 816

DAGGETT, B. H., Meatotomy . 197
Varicocele . . 357
Davis, Richard Harding, and Valet 65
Death-rates from Cancer and Consumption . . . . . . . . . . 892
Death, Sudden, After Fifty, Cause and Prevention of . . . . . . . . 88
Degeneracy . . 241
Dermatol in Treatment of Diarrhea, 575
Diabetes Mellitus, Treatment of . 846
Diarrhea, Subcutaneous Injection of Salt Solution in . . . 204
Subcutaneous Injection of Serum in 204
Diehl, A. E., Can Syphilis be Cured? 660
Digestion, Influence of Fatigue on . 303
Diphtheria Antitoxin . . . . . . 595
Patent on . . . 303
Disinfection, New Method of . . . . 688
Dislocations, Compound . . . . 414
Dooley, E. M., Colles's Fracture . . 571
Dowd, J. Henry, Injuries to the Urinary Organs . 335
Prevention of Urinary Infection . . . 737
Dressing, A Dry . . . 206
Drum-head, Rupture of not Incurable . . . 693
Dunham, Sydney A., Alcohol in Treatment of Mental and Nervous Diseases . 828
Sudden Death After Fifty . . 88
Durand, C. F., Chronic Follicular Prostatitis . . . . . . . . . . . . 118

ECZEMA and Washing of the Hands . . . . 696

Enterocolitis, Irrigation of the Bowel in . . . . . . . . . . . . . . . . 203

PAGE.

Epilepsy, Borax in . . . . . . . . . 596
Execution of a Woman . . 766
Eyes of School Children — Why They Should be Examined . . . . . . 495

FAKE JOURNAL . . . . 540
Flies and Water Supply in Spreading Disease . . . . 663
Foot Blisters, Incision in. . . . . 847
Forman, Eugene S., Memorial of . 919
Fractures, Treatment of T-shaped . . 54
Frederick, C. C., Necessity of Early Diagnosis of Malignant Disease of Pelvic Organs 655
Fronczak, F. E., Cholelithiasis (Translation) . . . . 832
Compound Dislocations 414
Frye, Maud J., Early Diagnosis of Uterine Cancer . . . . . . . . . 576

GARBAGE, Sewage, etc., Disposal of in Camps . . . . . . . 580
Gastric Carcinoma . . . . . . 18
Gibbs, John Blair, Memorial to 383
Gould, Miss Helen's Charities . . 137
Greene, Walter D., Typhoid Fever . 507
Grosvenor Library . . . 539
Gunshot Wounds by Modern Missiles . . . . . . . . . . . . . . . 1

HALL, A. L., Memorial of Eugene S. Forman . . 919
Harris, Peter C., Camps of Instruction . . . . . . . 492
Hartwig, Marcell, Some Points in Incipient Orthopedic Cases 732
Surgery of the Kidney . . 401
Headaches, Differential Diagnosis of, 695
Health of the Seventh Army Corps, 301
Heart Murmurs, Functional . . . . 846
Heat Stroke in Infants . . . . . . 600
Hemianopsia . . . . . . 513
Hernia, Radical Cure of Inguinal . . 418
Transplantation of Rectus Muscle in Inguinal . . . . 205
Heroin, Therapeutics of . . . . 761
Higgins, F. W., Hemianopsia . 513
Himmelsbach, G. A., Acute Nephritis in an Infant 809
Holocain versus Cocaine in Ophthalmic Surgery . . . . . . 841
Hospital Ship Missouri . . . . . . 64

PAGE.
Howe, Lucien, Why Eyes of School Children Should be Examined . 492
Hydrocele, Eversion of Tunica Vaginalis in . . . . . . . . . 296
Hyperchlorhydria . . . . . . . . 823
Hysteria and Brain Tumors . . . . 11
with Epilepsy . . . . . 502

ICHTHYOL in Diseases of the Eye . . . . . . . . . . . . . . 602

**Illustrations :**

Alopecia Areata (Spangenthal) . 278
A. R. Davidson . . . . . . . . 354
Barton, Clara . . . . . . . . . 61
Boardman, John . . . . 68
Buffalo General Hospital, 1858 . 269
1880 . 270
1898 . 272
Buffalo State Hospital . . . 275
Buffalo Woman's Hospital . . . 344
Charles H. Wilcox . . . . . . 587
Charles S. Hoyt . . . . . . 459
Consumption Annex . . . 342
Cronyn, John . . . . . . . . . 27
Dagenais, Alphonse . . . . . . 35
Earl, Wesley Clark . . . . . 940
Erie County Hospital . . 341
Facsimile Front Cover Buffalo Medical Journal, 1845 . . . . 350
First Buffalo Medical College . . 188
First Sisters of Charity Hospital . . . . . . . 266
Flandrau, Thomas M. . . . . . 142
Folwell, Mahlon B. . . . . . . 33
Forman, Eugene S. . . . . . 920
Frank Hamilton Potter . . . 356
F. R. Campbell . . . . . . 355
General Albert J. Meyer . . . 261
General Leonard Wood, facing . 697
George Henry Rohé . . . . 624
German Deaconess's Home . 346
Hemianopsia (Higgins), Figs. 1 and 2 . . . . . . . . 514
Figs. 3 and 4 . . . . . . 515
Hysteria with Epilepsy (Clark), Fig. 1 . . . . . . . . . . . 502
Fig. 2 . . . . . . . . . . . 503
Fig. 3 . . . . . . . . . 506
James N. Matthews . . . . . . 352
James O. Putnam . . . . . . 187
J. C. Greene . . . . . . . . 543
Judson B. Andrews . . . . . . 274
Julius F. Miner . . . . . . 353
Medical Department, Niagara University . . . . . . 263
Medical Staff, 65th Regiment, U. S. V. . . . . . . . . . . . . 590

PAGE.
Millard Fillmore . . . . 186
Morton, William T. G. . . . . . . 30
Potter, Milton Grosvenor . . . 194
Providence Retreat . . . . 340
Resuscitation of the Apparently Drowned, Fig. 1 . . . . . 934
Fig. 2 . . . . . 935
Fig. 3 . . . . . . . 935
Riverside Hospital . . . . 348
Second Buffalo Medical College, 190
Second Sisters of Charity Hospital . . . . . . . 268
State Medical Examining Board, 38
Tait, Lawson . . . . . . 938
Third Buffalo Medical College . 195

Immunes, Enlisting Regiments of . . 62
Inanition, Metabolism During . . 200
Indigestion in Infants . . . . . . . 81
Infant Constipation . . . . . . 290
Infants, Mortality Amongst . . . . 491
Insanity, Treatment of . . . . . . 852
Intestinal Anastomosis . . . . . . 53
Obstruction from Gall-stone . . 52
Pain of . . 250
Intussusception in Children . . . . 289
Iodoform Intoxication . . . . . . . 290
Iron, Absorption of . . . . . . . . 688

**Items :**

American Boarding House at Paris . . . . . . . . 807
British Army Medical Officer, Difficulties of . . . . 160
Bureau of the Medical Press 807
Christian Scientists in the Toils . . . . . . . . . . . . 880
Hatfield Prize . . . . . . . 159
Instruction of Soldiers in Hygiene . . . . . . . . 158
" Justice to Successful Practitioners and Students" . . . . 319
Kansas City Medical Index . . 808
Modern Vaccine Plant . . . . 480
New York Civil Service Examination . . . . . . . . . . 807
Patent on Diphtheria Antitoxin, 158
Reed & Carnrick . . . . . . . . 808
Sanitary Candle . . . . . 400
Santiago, Purification of . . . 160
Scott's Emulsion Vindicated . 954
St. Mary's Maternity and Infant Asylum . . . . . . . . . . 80
Stoddart Bros. . . . . . . . . . 954
Substitution . . . . . 808
William R. Warner & Company . . . . . . . . . . . 640

PAGE.

JACK, GEORGE N., Abnormalities of the Blood.—Etiology of Asthma . . . . . . . . . . 481
Jameson, Thomas, Tubercular Peritonitis with Report of Laparatomy Cases . . . . . . . . . . . . . 740
Jones, William B., Radical Cure of Inguinal Hernia . . . . . . . . . 418

KIDNEY, Movable; Its Treatment . . . . 122, 209
Surgery of . . . 210, 401
King, Clarence, Indigestion in Infants . . . . . . . . . . . . . . 81
Knapp, Louis H., Camp Water Supply . . . . . . . . . . . . . . . 592
Krauss, William C., Degeneracy . . 241
Hysteria and Brain Tumors, 11

LABORATORY of Neurology and Psychology at University of Pennsylvania . . . . . . 436
Landry's Paralysis . . . . . . . . . 596
Laparatomy, Management of Patients Before and After . . . . . 848

**Leading Articles:**

Alumni Reunion . . . . . . 609
Army Bill and the Medical Corps . . . . . . . . . . . 618
"Army Examinations and State License" . . . . . . . . . 704
Christian Science, A Sociological Study, 455
Fake . . . 622
General Leonard Wood . . . 697
Harold Frederic and Christian Science . . . . . . . . . . 377
Hospital Corps, 74th Regiment . . . . . . . . 534
Increase the Sanitary Inspectors . . . . . . . . . . . . 620
Medical Department of the Army and Its Critics, 127
Journalism . . . . . 57
Society of the State of New York . . . . 615
Medicine in Buffalo During 1898 . . . . . . . . . . . . 451
Military Camp Sanitation . . 218
Mobile Affair, The . . . . . 132
Outdoor Poor Relief in Buffalo. 699
Prevalence of Small-Pox . . 621
Resuscitation of the Apparently Drowned . . . . . . . . . 934
Sick and Wounded at Santiago, 55

PAGE.

State Control of Midwives . . 131
The Columbus Meetings . . . 930
The Impossible Leffingwell . . 764
The Legislature and the Profession . . . . . . . . . 702
Typhoid Fever in the Military Service . . . . . . . . . 854
War Investigation Commission, 297

Liquid Air as a Cautery . . . . . . 746
Lindheim, George W., Death of . . 220
On the Death of . . . . 381

**Literary Notes:**

American Medical Quarterly . . 951
Antikamnia Chemical Company, 399
Fetation and Parturition Chart . 8-6
Archives of Neurology and Psychopathology . . . 559
Provinciales de Medicine . . . . . . . . 559
Arlington Chemical Co . . . 952
Army Medical Examining Board, 240
Blood Chart . . . . . . . . . 79
Breitenbach's Company's Yearbook, 1899 . . . . . 480
Buffalo General Hospital . . . 952
Hospital Sisters of Charity . . . . . 952
State Hospital . . . . 951
Bulletin Harvard Medical Alumni Association . . . . 240
Canadian Journal of Medicine and Surgery . . . 80
Practitioner . . . . 400
Chirurgy in Medicine, Ambrose Paré . . . . . . . . . . . 479
Climate . . . . . . . . . . . 479
Defective Hearing in Relation to Shorthand Writing (Whitford) . . . . . . . 157
Dental Practitioner and Advertiser . . . . . . . . . . . 399
Gerrish's Anatomy . . . . . 805
Grosvenor Library . . . . . 400
Medical Department. 720
History of the Medical Department of Niagara University . 879
International Medical Annual for 1899 . . . 720
Medical Journal . 400
Medical Magazine . . . . 639
Review of Rhinology, etc . . 805
Jewett's Obstetrics . . . . . 480

PAGE.
Kansas City Lancet . . . 320
Medical Journal . . . 560
Maltine Company . . . . 157
Medical and Surgical Monitor . 80
Dial . . . . . . . . 480
Society County of Kings, History . . 806
Metaphysical Magazine 479
Michigan Health Resort Directory . . . 879
Mulford's Antitoxin Brochure . 399
Nature and Art (Hayden) . 158
North American Practitioner . . . . . . . . . . . 639, 880
Obstetrics . . . . . . . 640
Ohio Medical Journal . . . . 639
Patent and Trade-mark Laws . 479
Progressive Medicine . . 560
Saturday Evening Post (Philadelphia) . . . . 399
Saunders's Hand Atlases . . . 239
Ship's Doctor . . 79
Southern Medical Exchange . 239
Journal . . 560
St. Louis Medical Gazette . . 80
The Iris . . . . . . . . . . 952
The Jeffersonian . . . 880
Therapeutic Digest . . . 400, 805
Transactions, Medical Society State of New York . . . 78
Western Clinical Recorder . . 639

Loop, Ross George, Paranoia . . . 174
Loveland, Milk in the Sickroom Dietary . . . . . . . . . . . . . 321
Lucemia, Metabolism in . . . . . . 685
Lung, Surgery of . . 50
Lusk, Z. J., Address at Alumni Reunion . . . . . 747
A Plea for the Unification of Medical Societies . . . . . . . 881

MALT, Proteolytic Value of . . 602
Mann, M. D., Address by . . 610
McKelway, St. Clair, The Press and the Times . . . . . . . . . 857
Measles in the New-born . . . . . 288
Meatotomy . . . . . . . . . . . . 197

**Medical College, Hospital and Dispensary Notes:**

A Marine Hospital for Buffalo 944
Atlanta Medical College and Southern Medical College,
Brooks Memorial Hospital . . 632
Buffalo General Hospital . . . . . . . . 711, 795, 943
PAGE.
Buffalo State Hospital . . . . 795
Cancer Hospital . . . . . . . . 463
Consumption Hospital . . . 462
Cornell University Medical College . . . . 309, 548
Fresh Air Mission Hospital . 73
German Deaconess's Hospital 712
Hospital . . . 463, 711
Hospital Corps, 74th Regiment . . . . . . . 461
Medical College Consolidation . 228
Medico-Chirurgical College of Philadelphia . . . . 631, 794
New York Post-Graduate Medical School and Hospital . . . . . 146, 548, 711
Union of . . . . . . . 147
Northwestern University Woman's Medical School . 710
Ohio State Board of Registration . . . . . . . . . . . 463
Ozark Sanatorium . . . . . . 795
Riverside Hospital . . . . 712
Roosevelt Hospital . . . . . 548
Samaritan Hospital, Troy . 3[illegible]8
Sisters of Charity Hospital, Buffalo . . . . 308, 880
Skin and Cancer Hospital . . . 547
Southern Medical College Association . . . . . 462
St. Mary's Maternity and Infant Asylum . . . . . . . . . . 943
Syracuse University . . . . 796
University and Bellevue Hospital Medical College . . 863
of Buffalo . 464, 710, 796
of Buffalo and the State Laboratory . 146
of Edinburgh . . 549
Woman's Medical College of the New York Infirmary for Women and Children . . . . 943

Medical History of the County of Erie . . . . . . 25, 98, 182 260, 339, 424, 518, 586, 681
Journal, A Progressive . . 134
Journalism in Maryland . 457
Officers, Appointments of . 62
Societies, A Plea for the Unification of . . . . 881
Women and Medical Journals . . . 706
Medicine, Relation of to Civilisation . . . . . . . 902
Memorial to Dr. Joseph O'Dwyer . . 767
Military Surgery at Santiago . . . . 120
Milk in the Sickroom Dietary . . . 321
Millener, F. H., Nosophen and Antinosin in Middle Ear Disease . . . 332

PAGE.

**Miscellany** :

George H. Kinter . . . . . 953
Maria Muller . . . . . . . . 954
Samuel D. Gross Prize . . . 640
Stephen T. Lee . . . . . . . 952

Morphine Derivatives . . . . . . 423
Municipal Nuisances . . . . . . . 786
Restriction of Disease . . 161
Myxedema, Case of . . . . . . . 179

NAPHTHALENE, Physiological Action of . . . . . . 202
Navy, Assistant Surgeons in . . . 135
Nephritis, Acute, in an Infant . 809
Complicating Gastro-enteritis in Infants . . . 928
Nephropexy, Lumbar, without Suturing . . . . . . . 293
Nerves, Suturing of Divided . . . . 54
Nervous and Mental Diseases, Alcohol in Treatment of . . . . . . 828
Neurasthenia, Treatment of . . . 533
Newspaper Correspondent, First Honest . . . . . . . 383
Nicouline, Physiological Action of . 201
Nose-bleed, To Stop . . . . 206
Nosophen and Antinosin in Middle Ear Disease . . . . . . . . . 332

OBSTETRIC PRACTICE, Prophylaxis in . . . . . . . 886

**Obituary** :

Bailey, William H. . . . . . . 69
Boardman, John . . . . 67
Bowen, Major H. C. . . . . . 138
Burr, Franklin E. . . . . . . . 225
Clark, C. C. P . . . . . . . . 547
Crawford, A. H . . . . . . 544
Earl, Wesley Clark . . . . . . 937
Ethridge, James Henry . . . . 626
Flandrau, Thomas M. . . . . . 139
Forman, Eugene S. . 225
Greene, J. C . . . . . . . . . 543
Gleason, S. O . . . . . . . 790
Griffith, Benjamin M. . . . . . 304
Haberstro, Joseph . . . . . . . 69
Hamilton, John B. . . . . . 545
Haynes, Francis L . . . . . . 386
Hoyt, Charles S . . . . . . . 459
Johnson, B. R . . . . . . . . 546
Joslyn, Henry . . . . . . . . 627
Lincoln, Nathan Smith . . . 304
Mastin, Claudius Henry . . . . 305

PAGE.

Owen, Abraham M. . . . . . 224
Parke, Hervey Coke . . . . . 626
Parker, L. P. L . . . . . . . . . 546
Pepper, William . . . . . . . . 142
Pickett, John H. . . . . . . 141
Powers, Harry A. . . . . . . . . 385
Pratt, H. De V . . . . . . 546
Rohé, George Henry . . . . . 623
Showerman, James M. . . . . 941
Stillman, Edwin M . . . . . . 461
Struthers, Sir John . . . . . 627
Tait, Lawson . . . . . . . 937
Taylor, Charles Fayette . . . . 789
Turner, Robert C . . . . . . 386
Waring, Col. George E. . . . . 320
Willard, A. E . . . . . . . . . 386

Ocular Hygiene in Public Schools . 561
Optic Neuritis, Localization Value of, etc . . . . . . . . . . . 843
Orthoform, "New" . . . . . . . . 532
Orthopedic Cases, Some Points in Incipient . . . . 732
Osteomyelitis Following Traumatism, 207
Oxygen, Absorption of by the Lungs 200

PAN-AMERICAN Exposition and American Medical Association . . . 705
Paralysis, Phrenic, with Transposition of Heart . . . . . . 97
Paranoia . . . . . . . . . . . . 174
Park, Roswell, Gunshot Wounds by Modern Missiles and Their Treatment . . . . . . . . . 1
Pelvic Diseases, Diagnosis and Treatment of Suppurative . . 251
Organs, Early Diagnosis of Malignant Disease of . . 655
Pen Pictures of the Alumni Meeting, 701
Pensions, Annual Report of Commissioner of . . . . 223
in the War with Spain . . 63
Pepto-Mangan (Gude) in Surgical Convalescence . . . . . 293
Peritonitis, Tubercular; with Report of Cases Apparently Cured by Laparatomy . . . . 740

**Personal** :

Wood, Brigadier-General Leonard . . . 60
Reed, Charles A. L.; McMurtry, L. S. . . . . . 66
Draper, William H.; Kenerson, Vertner; Knowles, Charles P.; Ingraham, Henry D. . . . . 67

PAGE.
Mathews, Joseph McDowell . . 138
Jenkins, F. T.; Uhlman, Julius; Wilson, Nelson W.; Downer, Louise; Renner, W. Scott 139
Bentz, H. G.; Wilson, Nelson W.; Le Breton, Prescott . . . 224
Krauss, William C. . 303
Clark, Edward; May, William B.; Peterson, Frederick . . 304
Knowlton, Charles Benjamin . . 320
Briggs, A. H.; Meade, Harry A.; Ruffner, E. L. . . 384
Jelks, James T.; Trowbridge, Grosvenor R.; Bryant, Joseph D.; Crowe, T. M. . 384
Thomas, George G.; Wilson, Nelson W.; Bruso, C. F.; Metcalfe, F. T.; McKee, T. H.; Crockett, Montgomery A.. 385
Earl, Wesley C.; Webster, David; Goltra, John N.; Vandenbergh, Bina P.; Knowles, Charles P. . . 458
Stackhouse, O. C.; Grant, John H.; Millener, F. H. . . 541
Mountain, W. H.; Ill, Edward J.; Fowler, George R. . . . . 542
Simmons, George H. 622
Stocker, George B.; Hoyer, B. P.; Himmelsbach, G. A.; Aldrich, N. W.; Mansperger, W. H.; Rooth, H. C. . . . . 623
Dorr, Samuel G. . . 706
Thornbury, F. J.; Fowler, George Ryerson; Suiter, A. Walter; Wende, Grover W.; Rogers, Benjamin F.; Williams, Herbert U. . 707
Jewett, Charles; Reamy, Thaddeus A. . . . . . . 787
Gaertner, Wilhelm; Clinton, Marshall; Macbeth, A. H.; Johnson, Burt C.; Putnam, B. H.; Wall, Charles A.; House, William . . 788
Dowd, J. Henry; Bissell, William G.; Meyer, Edward J . 789
Reed, Charles A. L.; Lewis, Daniel; Crego, Floyd S.; Flagg, J. D. . . 859
Allen, A. B.; Burnham, N. L.; Currier, A. F.; Krauss, William C.; Deaver, John B.; Cott; Geo. F.; Palmer, Geo. S. . . . . . . . 860
Pain, Horace M.; Wilson, Nelson W.; Breuer, Max C.; Starr, M. Allen; Richmond, Nelson G. . . . . . . . . . . 936

PAGE.
Abbott, Frank W.; Lewis, F. Park; Hubbell, Alvin A. . . 937

Pneumonia, Ether . . . . . . . 54
in Children . . . . . 689
Phthisis, Murphy's Method of Treating . . . . . . . . . . . 208
Politics and Education . . . . . 784
Popliteal Artery Wounds . . . . . 53
Potter, William Warren, Address at Alumni Reunion . 750
Medical History of the County of Erie 25, 98, 182, 260, 339, 424, 518, 586, 681
Price, Joseph, Diagnosis and Surgical Treatment of Suppurative Pelvic Diseases . . . . . . . . . . . 251

**Progress in Medical Science:**

Genito-urinary and Syphilitic Diseases, by B. H. Daggett . . 122, 209, 293, 596, 848
Medicine, Pathology and Therapeutics, by Albert T. Lytle . . . . . 288, 595, 846
Ophthalmology, by A. A. Hubbell . . . 211, 600, 841
Pediatrics, by Maud J. Frye . 203, 598, 689, 928
Physiological Chemistry, by John A. Miller . . 200, 370, 685
Surgery, by John Parmenter and Nelson W. Wilson 50, 120, 205

Prostatitis, Chronic Follicular . . . 118
Protargol in Ophthalmia Neonatorum 292
Ophthalmic Practice . . 211
Protonuclein in General Practice . . 446
Pryor, J. H., Relative Death-rates from Cancer and Consumption . . 892
Psoas Abscess in Women . . . . . 326
Public Parks and Public Health, 696, 851
Pupil, The, in Disease . . . . . . 213

QUININE Industry on the Island of Java . 300
To Mask Taste of . . 291

RECTUM, Cancer of . . . . . 54
Treatment of Prolapse of . . . . . . . . 207
Red Cross and Clara Barton . . . . 61

### Reviews :

PAGE.

Addresses and Papers in Veterinary Medicine (Cornell) . 876
Allen : Practitioner's Manual . . 554
Annual Report of Michigan State Board of Health, 1896 . . . 396
Annual Report State Board of Health of Pennsylvania, 1897, 871
Bacon : Manual of Otology 313
Bailey : Accident and Injury . 551
Baldy : American Text-Book of Gynecology . . . . . . 471
Barlow : Day Dreams of a Doctor . . . . . . . . . . 154
Baruch : Hydrotherapy . . . 553
Beevor : Diseases of the Nervous System . . . 633
Bianchi : The Phonendoscope . 803
Bishop : Diseases of the Ear, etc. . . . . . . . . . . . . 803
Bocquillon-Limousin : Formulaire des Médicaments Nouveaux pour 1899 . . . 718
Buck : Diseases of the Ear . . 716
Bulkley : Manual of Diseases of the Skin . . . . . . . 393
Bushong : Modern Gynecology, 235
Butler : Materia Medica, Therapeutics and Pharmacology 474
Cabot : Examination of the Blood . . . . . . . . 316
Clouston : Lectures on Mental Diseases . . . . . . 557
Coleman : Syllabus of Materia Medica . . . . . . . 872
Crile : An Experimental Research into Surgical Shock . . 946
DaCosta : Manual of Modern Surgery . . . . . . . . . 311
Davenport : Diseases of Women, 558
De Schweinitz : American Text-Book of Diseases of the Eye, Ear, Nose and Throat . 866
De Schweinitz : Diseases of the Eye . . . . . . . . . . . 878
Donders : Anomalies of Refraction . . . . . 870
Dorland : Pocket Dictionary . . 476
Dudley : Principles and Practice of Gynecology . . . . . 314
Dunham : Normal and Morbid Histology . . . . 717
Edinger-Hall : Anatomy of the Central Nervous System . 877
Egbert : Hygiene and Sanitation 154
Einhorn : Diseases of the Stomach . . . . . . . . . . 311
Fullerton : Obstetric Nursing 877
Gerhard : Sanitary Engineering, 155

PAGE.

Gerrish : A Text-book of Anatomy . . . 944
Gottheil : Atlas of Skin Diseases . . . . . . . 231
Gottheil : Treatment of Skin Cancers . . . . . 309
Gould : American Year-book of Medicine and Surgery, 637
Pocket Medical Dictionary . . . . . . . 637
Grandin-Jarman : Practical Obstetrics . . . . . . 872
Green : Pathology and Morbid Anatomy . . . . . . . . . 472
Griffith : Care of the Baby . . . 396
Groff : Handbook of Materia Medica. . . . . . . . . . 395
Grünwald : Atlas of Diseases of the Larynx . . . . . 312
Haig : Diet and Food . . . . . 716
Hardaway : Manual of Skin Diseases . . . . . . . . 309
Hamilton : Lectures on Tumors, 313
Hansell : Muscular Anomalies of the Eye . . . . 870
Hare : Practical Diagnosis . 715
Hare : Progressive Medicine 802
Hare : Text-Book of Practical Therapeutics . . . . . . 470
Hartridge : Refraction of the Eye . . . . . . . . . 557
Hayden : Venereal Diseases . . 803
Heitzmann : Urinary Analysis and Diagnosis . . . . . . . 875
Herman : Diseases of Women . 152
Herrick : Railway Surgery . . 797
Hinsdale : Acromegaly . . . . 557
Hirst : Text-Book of Obstetrics . . . . . . . . . 712
Hoffman : Atlas of Legal Medicine . . . . . 237
Hollopeter : Hay Fever . . . . 76
Index-Catalogue Library Surgeon-General's Office . 556
International Clinics, Eighth series, Vol. II., 153 ; Vol. III., 473 ; Vol. IV . . . . 868
International Medical Annual and Practitioner's Index . . 875
Jakob : Atlas of Clinical Investigation . . . . . . . 156
Jewett : Practice of Obstetrics . 864
Johns Hopkins Hospital Reports, Vol. VII . . . . . . . . . 230
Keen : Surgical Complications of Typhoid Fever . . 389
Kelsey : Treatment of Hemorrhoids . . . . . . . . . . 313
Klein : Elements of Histology . 476
Lambert : Choleraic Diarrhea . 76

PAGE.
Landis-Wells: Compend of Obstetrics . . . . . 876
Lane: Cleft Palate . . 798
Lazarus-Barlow: Manual of General Pathology . 391
Loomis-Thompson: System of Practical Medicine . . . 234
Manson: Tropical Diseases . . 236
McFarland: Pathogenic Bacteria, 393
Medical News Visiting List, 1899, 397
Medical Record Visiting List, 1899 . . . . . . . 477
Menninger: Diagnosis of the Urine . . . . . . . . . 948
Miller: Student's Histology . . 552
Moore: Orthopedic Surgery . 75
Morris: Diseases of the Skin . 475
Morris: Essentials of Materia Medica, etc . . . . . . 871
Morris: Human Anatomy . . . 469
Morris: Renal Surgery . . 801
Mracek: Atlas of Syphilis and Venereal Diseases . . . 310
Norris-Oliver: System of Diseases of the Eye . 147
Ohlemann: Ocular Therapeutics, 871
Osler: Practice of Medicine . . 800
Park: An Epitome of the History of Medicine . . . 948
Physician's Visiting List (Lindsay & Blakiston), 1899 . . . . 397
Pilcher: Treatment of Wounds, 797
Playfair: Treatise on Midwifery, 555
Powell: Pocket Medical Formulary . . . 717
Presbyterian Hospital Reports, Vol. III . 236
Public Health Reports, Marine Hospital Service, Vol. XII . 233
Purdy: Practical Urinalysis 638
Reese: Text-Book of Medical Jurisprudence and Toxicology, 233
Report of Commissioner of Education, 1896 97, Vol. I , Part 1, 316
Report of Health Department of San Francisco . 237
Report of State Board of Health of New York, 1896 394
Roberts: Orthopedic Surgery . 799
Rose: Manual of Surgery 468
Sajous: Annual and Analytical Encyclopedia of Practical Medicine, Vol. II., 636; Vol. III . . . 874
Sanger: History of Prostitution, 633
Schafer: Essentials of Histology, 477
Schamberg: Diseases of the Skin . . . . . . . 395
Scheppegrell: Electricity in Diseases of the Nose, etc. . . 394

PAGE.
Scott: The Sexual Instinct . . 714
Shaeffer: Essays on Orthopedic Surgery . . . . . . 233
Simon: Manual of Chemistry . 555
Slater: Atlas of Bacteriology . 713
Smart: Handbook for the Hospital Corps of the Army . . 230
Starr: American Text-Book of Diseases of Children . . 473
Stedman: Twentieth Century Practice, Vol. XIV., 74; Vol. XV., 229; Vol. XVII . . . . 635
Stengel: Text-book of Pathology 949
Stevenson: Wounds in War 228
Stewart: Manual of Physiology . . . . . . . . . . 876
Stimson: A Practical Treatise on Fractures and Dislocations . . 947
Taylor: Diseases of Children . . 392
Taylor: Manual of the Practice of Medicine . . . 718
Thompson: Diseases and Injuries of the Conjunctiva . . 476
Thompson: Examination and Treatment of Sick Children . 475
Thorington: Retinoscopy . . . 860
Three Thousand Questions on Medical Subjects . . . 872
Touatre: Yellow Fever . . . . 75
Transactions: American Association of Obstetricians and Gynecologists . . . . 76
American Gynecological Society . . . . . . 869
American Laryngological Association . . . . . . . 155
American Surgical Association . . . . . . . . . . 555
Association of American Anatomists . . . . . . . 315
Medical Association of Central New York . . . . . 234
Medical Society of the State of New York . . . . 310
Ohio State Medical Society, 634
Southern Surgical and Gynecological Society . . . . 153
Texas State Medical Association . . . . . . . . . 878
Wisconsin State Medical Society . . . . . . 634
Tyson: Manual of Practical Diagnosis . . . . . . . . . . 312
Van Valzah: Diseases of the Stomach . . . . . . 232
Vecki: The Pathology and Treatment of Sexual Impotence . . . 948
Vierordt: Medical Diagnosis . 635
White: Materia Medica, etc 869

PAGE.

Williams: Diseases of Infancy and Childhood . . . . 556
Williams: Manual of Bacteriology . 873
Witthaus: Urinalysis and Toxicology . . . . . . 874
Wyeth: Text-Book on Surgery, 150
Year-Book of New York State Reformatory 395
Zuckerkandi: Atlas of Operative Surgery . 396

Rheumatism, Hydrochloric Acid in 846
Pyrosal in Acute . . . 847
Rhinolith . . 371
Roe, John O., Relation of Medicine to Civilisation 902
Rogers, Benjamin F., Ocular Hygiene in Our Public Schools . . . . . 561

SALICYLIC ACID, Behavior of in the Organism . . . . . . 201
Salol . . . . . . . 291
Sanitary Supervision During the Exposition . . . . . . . . 785
Sanitation at Santiago . . . 299
Santiago, Explanation of Difficulties Surrounding Campaign of . . . . . . 133
Mail Facilities at . . . 378
Sarcoma, Serum Treatment of . . 53
Scarlatina . . . . . . . . . 568
Seat of Life in Man . . . . . 372
Senn, Lieutenant-Colonel Nicholas, Criticisms by . . . . . . 220
Sickness in the Army . . . . 538
Sleep in the Treatment of Disease . 694
Small Pox in Western New York . . 420

**Society Meetings:**

Alumni Association, Medical Department, Niagara University, 863
American Academy of Medicine . . . . . . 628, 862
American Academy of Railway Surgeons . 227
American Association of Obstetricians and Gynecologists . . . . . . 70, 226, 792
American Climatological Association . 794
American Dermatological Association . 862
American Electro-Therapeutic Association . 71, 146, 550, 862
American Medical Association . . . . . . . . . . . . 708, 941

PAGE.

American Medical Editors' and Publishers' Association 791
American Medico-Psychological Association . 861
American Microscopical Society . 227
American Neurological Association . . . 941
American Public Health Association . . 145, 388
Buffalo Academy of Medicine . 72, 142, 226, 306, 388, 467, 550-551, 629, 709, 793, 861, 942
Cincinnati Obstetrical Society . 631
Esculapian Club . . 308, 389, 550, 631, 862
Iowa State Association of Railway Surgeons 308
Lake Erie Medical Society . 388, 631, 943
Medical and Chirurgical Faculty of Maryland . . . 790
Medical Association of Central New York . . . 145, 228
Medical Society of the County of Chautauqua 464, 942
Medical Society of the County of Chemung . . 861
Medical Society of the County of Erie . . . . 465, 861
Medical Society of the State of New York . . . . . . 307, 464
Medical Union . 549
Mississippi Valley Medical Association . 227, 307, 792, 942
National Association of Homeopathic Medical Examiners . . 861
National Confederation of State Medical Examining and Licensing Boards . 708, 862
New York State Association of Railway Surgeons 227, 388
New York State Medical Association 145, 228
New York State Medical Association, Third District Branch . 791, 860
Niagara Falls Academy of Medicine . 71, 549, 793, 942
Ninth International Ophthalmological Congress . . . . . 631
Ohio State Medical Society . . 860
Pan-American Medical Congress . . . 387
Rectal Specialists . . . . . . . 794
Roswell Park Medical Club . . 465
Sanatory Club of Buffalo . 465
Section on Materia Medica, Pharmacy and Therapeutics, A. M. A . . . . . . . . . . . 791

PAGE.
Seventh International Congress Against Use of Alcoholic Liquors . . 630
Sixth International Otological Congress . . . 549
Southern Surgical and Gynecological Association 146, 307, 466
The One Hundred Year Club . 707
Third International Congress for Gynecology and Obstetrics . . 791
Tri-State Medical Association . 630
Tri-State Medical Society . . . 227
Tuberculosis Congress . . . 793
Western Ophthalmologic and Oto-laryngologic Association . . . 709
Western Surgical and Gynecological Association . . . . . 308

**Society Proceedings :**

Buffalo Academy of Medicine 41, 216, 435, 757, 921
Medical Association of Central New York . . . . . . . 360
Medical Society of the County of Chautauqua . . . 48
Medical Society of the County of Erie . 44, 525, 594, 923
Sanatory Club of Buffalo . . . 523

Soldiers, Physical Condition of on Discharge . . . . . . . . . . 221
Spangenthal, Alopecia Areata . . 276
Speakers, A Classification of . . 759
State Examination Statistics . . . . 780
Statistics of the War with Spain . . 536
Sternberg, Surgeon-General George M., Report of . . . . 439
Stockton, Charles G., Phrenic Paralysis with Transposition of Heart . 97
Stomach, Removal of . 52
Streets, Unhealthy Nature of Torn Up . . . . . . . . . . 135
Substitution . . . . . . . . . . 607
Sugar as a Food . . . . . . . . . 687
Supplies in Cuba . . . . . . . . . 379
Surgical Intervention . . . . . . 382
Notes . . . . . . . . . 208
Sins . . . . . . . . . . 208
Syphilis, Can it be Cured ? . . . . . 660
Experiences in Treatment of . . . . . . . . . 295
Hypodermic Use of Mercury in . . 695
Later Form of Hereditary . 207
of the Stomach . . . . . 288
Stages and Forms of . . . 596

PAGE.
Syphilitic Eruptions . . . . . . . . 850
Hemiplegia . . . . . . . . 848

TAIT, LAWSON, " Conservative Surgery " as Applied to the Uterus and Appendages . . . 721
Tannigen . . . . . . . . 290
Taylor, W. G., Address at Alumni Reunion . . . . . . 748
Prophylaxis in Obstetrical Practice . . . . . . . . . . . . 886
Therapeutic Formulæ . . . . . . 291
Throat, Bacilli in . . . . . . . . . 331
Thyroid Gland, Chemistry and Action of . . . . . . . . . . 686
Tonsilitis, Acute Follicular . . . . 199
Treatment of Dysentery . . . . . . 929
Trowbridge, Grosvenor R., Cholera Infantum . . . . . . . . . . 409
Tubercular Bacilli on Grasses . . . 595
Tuberculosis, Creasote in . . . . . 289
Typhoid Fever . . . . . . . . . 507
Military Commission to Investigate Causes of . . . 136
Patients, Removal of from Field Hospitals . . . . 137
The Banana in . . . . . . 215

UHLMAN, JULIUS, Gastric Carcinoma . . . . . . . . . 18
University of Buffalo Commencement . . . . . . . . . . 768
Uric Acid in the Saliva . . . . . . 202
Urinary Infection—Its Prevention . 737
Organs, Injuries to . . . . 335
Urine, Casts in . . . . . . . 847
Changes in, Produced by Turkish Baths . . . . . 687
Estimation of Indican in . . 688
Glucose in . . . . . . . . 686
of Healthy Infants . . . . . 598
Reaction of Quinine on . . . 847

VACCINATION, Duration of Immunity After . . . 126
Vaginal Versus Suprapubic Celiotomy . . . . . . . . . . . 641
Van de Warker, Ely, Psoas Abscess in Women . . . . . . . . . . 326
Van Reypen, W. K., Surgeon-General U. S. Navy, Report of . 287
Varicocele . . . . . . 357
Veeder, M. A., Relative Importance of Flies and Water Supply in Spreading Disease . . . . . . . . 663

PAGE.

WARING, COL. GEORGE E., Memorial Fund to . . . . 383
Water Supply at Montauk Point . . 138
Wende, Ernest, Municipal Restriction of Disease . . . 161
Wende, Grover W., Electricity in Acne Vulgaris and Rosacea . . . 254
Wheeler, General Joseph Before the War Investigation Commission . . 381
Williams, H. T., Advantages of Vaginal over Suprapubic Celiotomy . . 641

PAGE.

Wood, General Leonard . . . . . 535
Speech of at Union League Club Dinner . . . . 536
Women; What They Did in the War, 539

YELLOW FEVER Investigation Commission . . . . . . . . . 538
Young, A. A., Carcinoma of the Testicle . . . . . 23
Scarlatina . . . . 568

